# A case of APMPPE-like panuveitis presenting with extensive outer retinal layer impairment following COVID-19 vaccination

**DOI:** 10.1186/s12886-023-02978-2

**Published:** 2023-05-24

**Authors:** Yo Ogino, Kenichi Namba, Daiju Iwata, Kayo Suzuki, Kazuomi Mizuuchi, Miki Hiraoka, Nobuyoshi Kitaichi, Susumu Ishida

**Affiliations:** 1 Department of Ophthalmology, Faculty of Medicine and Graduate School of Medicine, Hokkaido University, N-15, W-7, Kita-Ku, Sapporo, 060-8638 Japan; 2 Department of Ophthalmology, Health Sciences University of Hokkaido, Sapporo, Japan

**Keywords:** COVID-19 vaccination, Acute posterior multifocal placoid pigment epitheliopathy, Multimodal imagings, Steroid pulse treatment

## Abstract

**Background:**

Vaccination against the worldwide pandemic coronavirus disease 2019 (COVID-19) is underway; however, some cases of new onset uveitis after vaccination have been reported. We report a case of bilateral acute posterior multifocal placoid pigment epitheliopathy-like (AMPPE-like) panuveitis after COVID-19 vaccination in which the patient’s pathological condition was evaluated using multimodal imaging.

**Case presentation:**

A 31-year-old woman experienced bilateral hyperemia and blurred vision starting 6 days after her second inoculation of the COVID-19 vaccination. At her first visit, her visual acuity was decreased bilaterally, and severe bilateral anterior chamber inflammation and bilateral scattering of cream-white placoid lesions on the fundus were detected. Optical coherence tomography (OCT) showed serous retinal detachment (SRD) and choroidal thickening in both eyes (OU). Fluorescein angiography (FA) revealed hypofluorescence in the early phase and hyperfluorescence in the late phase corresponding to the placoid legions. Indocyanine green angiography (ICGA) showed sharply marginated hypofluorescent dots of various sizes throughout the mid-venous and late phases OU. The patient was diagnosed with APMPPE and was observed without any medications. Three days later, her SRD disappeared spontaneously. However, her anterior chamber inflammation continued, and oral prednisolone (PSL) was given to her. Seven days after the patient’s first visit, the hyperfluorescent lesions on FA and hypofluorescent dots on ICGA partially improved; however, the patient’s best corrected visual acuity (BCVA) recovered only to 0.7 OD and 0.6 OS, and the impairment of the outer retinal layer was broadly detected as hyperautofluorescent lesions on fundus autofluorescence (FAF) examination and as irregularity in or disappearance of the ellipsoid and interdigitation zones on OCT, which were quite atypical for the findings of APMPPE. Steroid pulse therapy was performed. Five days later, the hyperfluorescence on FAF had disappeared, and the outer retinal layer improved on OCT. Moreover, the patient’s BCVA recovered to 1.0 OU. Twelve months after the end of treatment, the patient did not show any recurrences.

**Conclusions:**

We observed a case of APMPPE-like panuveitis after COVID-19 vaccination featuring some atypical findings for APMPPE. COVID-19 vaccination may induce not only known uveitis but also atypical uveitis, and appropriate treatment is required for each case.

**Supplementary Information:**

The online version contains supplementary material available at 10.1186/s12886-023-02978-2.

## Background

Coronavirus disease 2019 (COVID-19), which began at the end of 2019, is a worldwide pandemic, and periodic inoculation of messenger RNA vaccines for COVID-19 has been used to prevent the disease by triggering an antibody response. Although these vaccines are highly effective, adverse events in various organs, including uveitis, have been reported.

We report a case of bilateral acute posterior multifocal placoid pigment epitheliopathy-like (APMPPE-like) panuveitis after COVID-19 vaccination in which the pathological condition was evaluated using multimodal imaging.

## Case presentation

A 31-year-old woman experienced a headache and a slight fever the day after her second inoculation of the COVID-19 vaccination (Comirnaty, Pfizer-BioNTech). Incidentally, she had no adverse reactions after her first vaccine (Comirnaty) other than a fever lasting 2 days. She recovered from the headache and fever the next day; however, 4 days later, she experienced bilateral hyperemia and blurred vision. Then, she visited an ophthalmology clinic where bilateral decreased visual acuity and panuveitis were observed. She was referred to our hospital 2 days later. She had no particular past history and family history. In addition, she did not show any neurological symptoms and skin lesions.

**Fig. 1:**
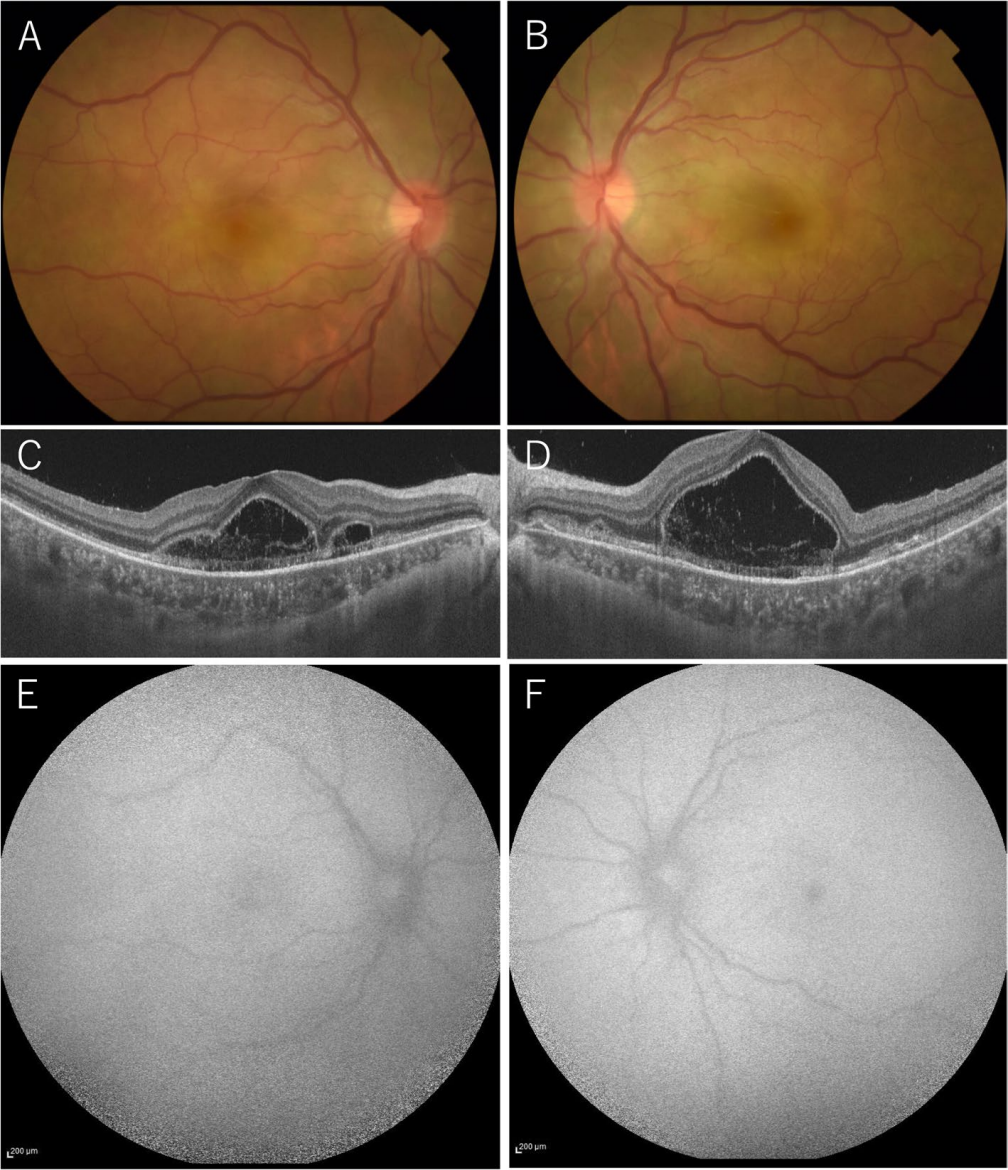
Fundus photographs and OCT and FAF findings from the initial visit. Redness of the optic disc and a scattering of cream-white placoid lesions were observed OD **A** and OS **B**. OCT revealed SRD and choroidal thickening OD **C** and OS **D**. No obvious abnormalities were detected with FAF OD **E** or OS **F**

**Fig. 2:**
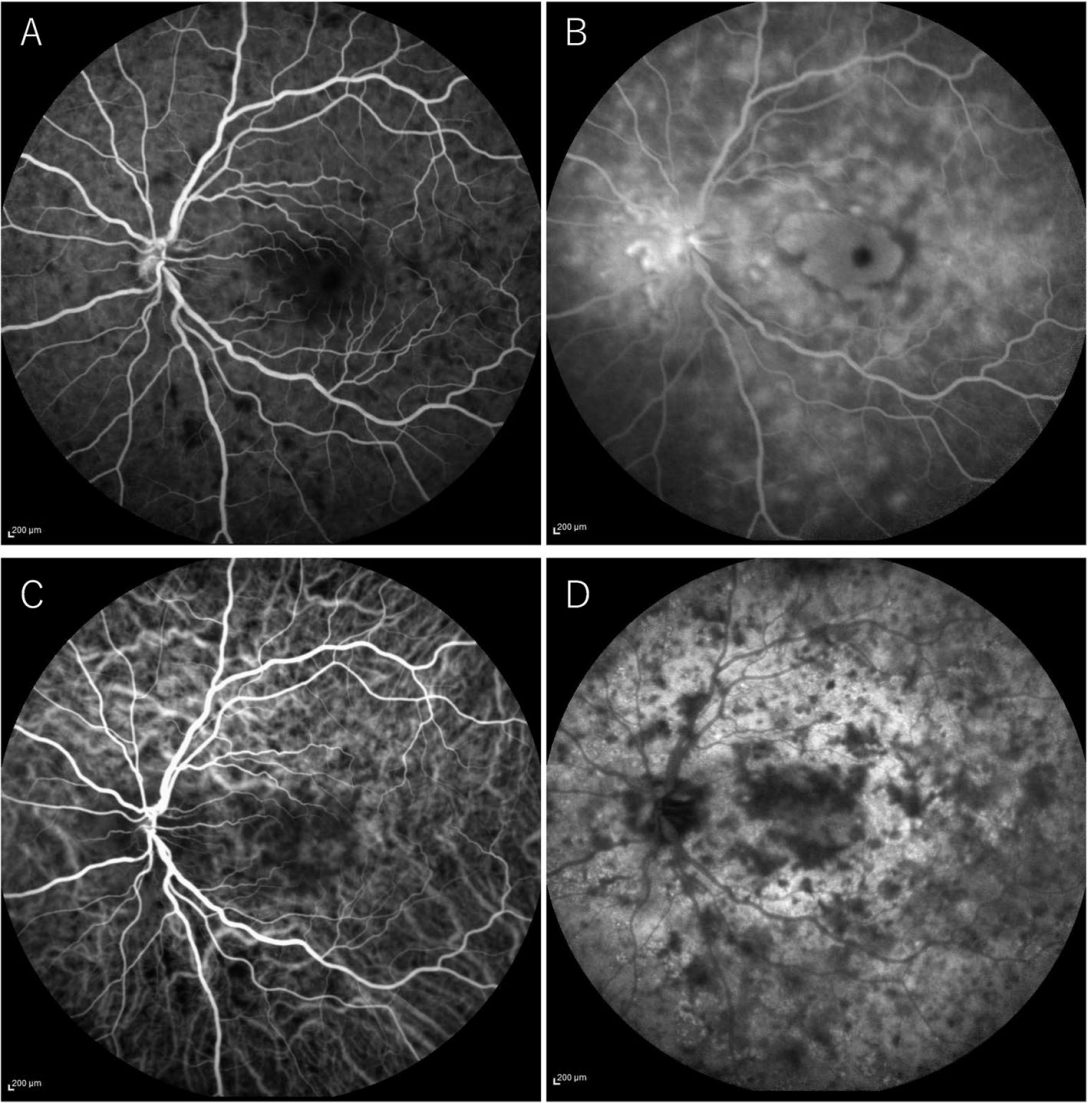
FA and ICGA of left eye in the early and late phases from the initial visit. FA revealed hypofluorescence in the early phase **A** and hyperfluorescence in the late phase **B** corresponding to cream-white lesions. ICGA revealed sharply marginated hypofluorescent dots of various sizes throughout the mid-venous **C** and late phases **D**

At her first visit, her best corrected visual acuity (BCVA) was 0.2 in her right eye (OD) and 0.1 in her left eye (OS) with mild myopia. The intraocular pressure was normal in both eyes (OU). Slit-lamp examination revealed bilateral ciliary injections and bilateral anterior chamber inflammation (2 + flare and 3 + cells) with fine keratic precipitates and anterior vitreous cells. Fundus examination revealed redness of the optic disc and a scattering of cream-white placoid lesions OU. Optical coherence tomography (OCT) revealed serous retinal detachment (SRD) and choroidal thickening OU. Fundus autofluorescence (FAF) examination did not reveal any abnormal findings (Fig. 1). Fluorescein angiography (FA) revealed hypofluorescence in the early phase and hyperfluorescence in the late phase corresponding to the cream-white lesions OU. Indocyanine green angiography (ICGA) revealed sharply marginated hypofluorescent dots of various sizes throughout the mid-venous and late phases OU (Fig. 2), which differed from typical findings seen in Vogt–Koyanagi–Harada (VKH) disease (i.e., vaguely marginated hypofluorescent small dots scattered throughout the fundus). Both blood analysis and urine analysis showed normal results, including a negative T-SPOT.TB, and cerebrospinal fluid examination did not show pleocytosis. Positron emission tomography–computed tomography also showed no abnormality. We did not request a brain MRI due to the absence of any neurological symptoms (including headache). The patient was suspected of having APMPPE with relatively severe anterior chamber inflammation; therefore, she was observed without any medications. Three days later, her SRD disappeared spontaneously.

**Fig. 3:**
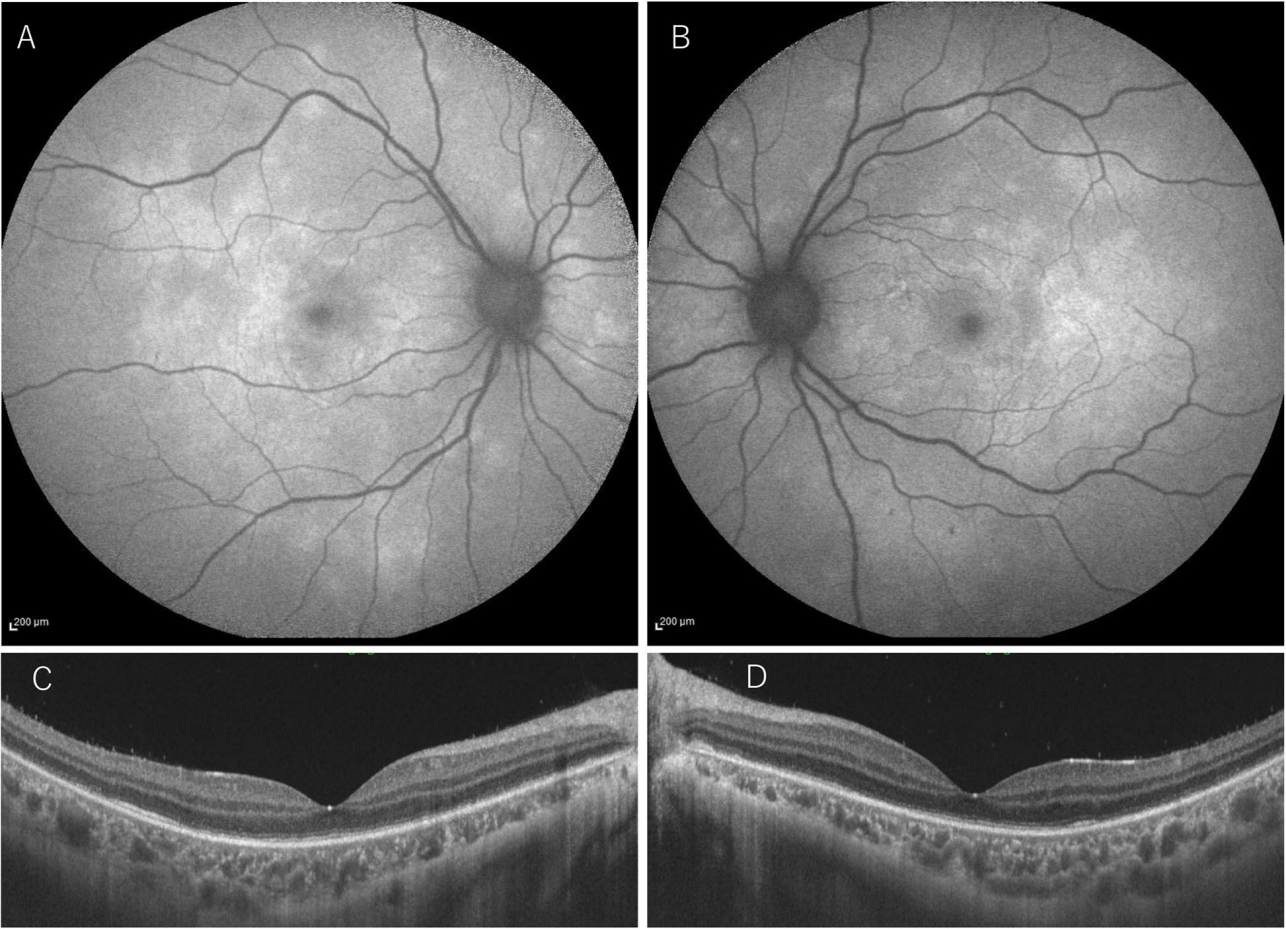
FAF and OCT 7 days after the first visit. The development of hyperautofluorescent lesions OD **A** and OS **B** was detected with FAF. Impairment of the outer retinal layer (irregularity in or disappearance of the ellipsoid and interdigitation zones) OD **C** and OS **D** was observed on OCT

However, her anterior chamber inflammation persisted, and her subjective symptom (darkening of vision) did not improve. At the request of the patient and her family, she received prednisolone (PSL; 100 mg for 3 days following 80 mg for 2 days). Seven days after the first visit, the hyperfluorescent lesions on FA and the hypofluorescent dots on ICGA partially improved; however, the patient complained that her vision was becoming darker. Her BCVA improved only to 0.7 OD and 0.6 OS. Additionally, FAF examination revealed the development of hyperautofluorescent lesions, and OCT revealed impairment of the outer retinal layer showing irregularity in or disappearance of the ellipsoid and interdigitation zones (Fig. 3). These findings were seen more broadly than the scattering white spots. According to these findings, we considered that the inflammation was exacerbating that required additional anti-inflammatory treatments. Therefore, steroid pulse therapy (1,000 mg of methylprednisolone daily for 3 days) was performed, followed by 60 mg of oral PSL.

**Fig. 4:**
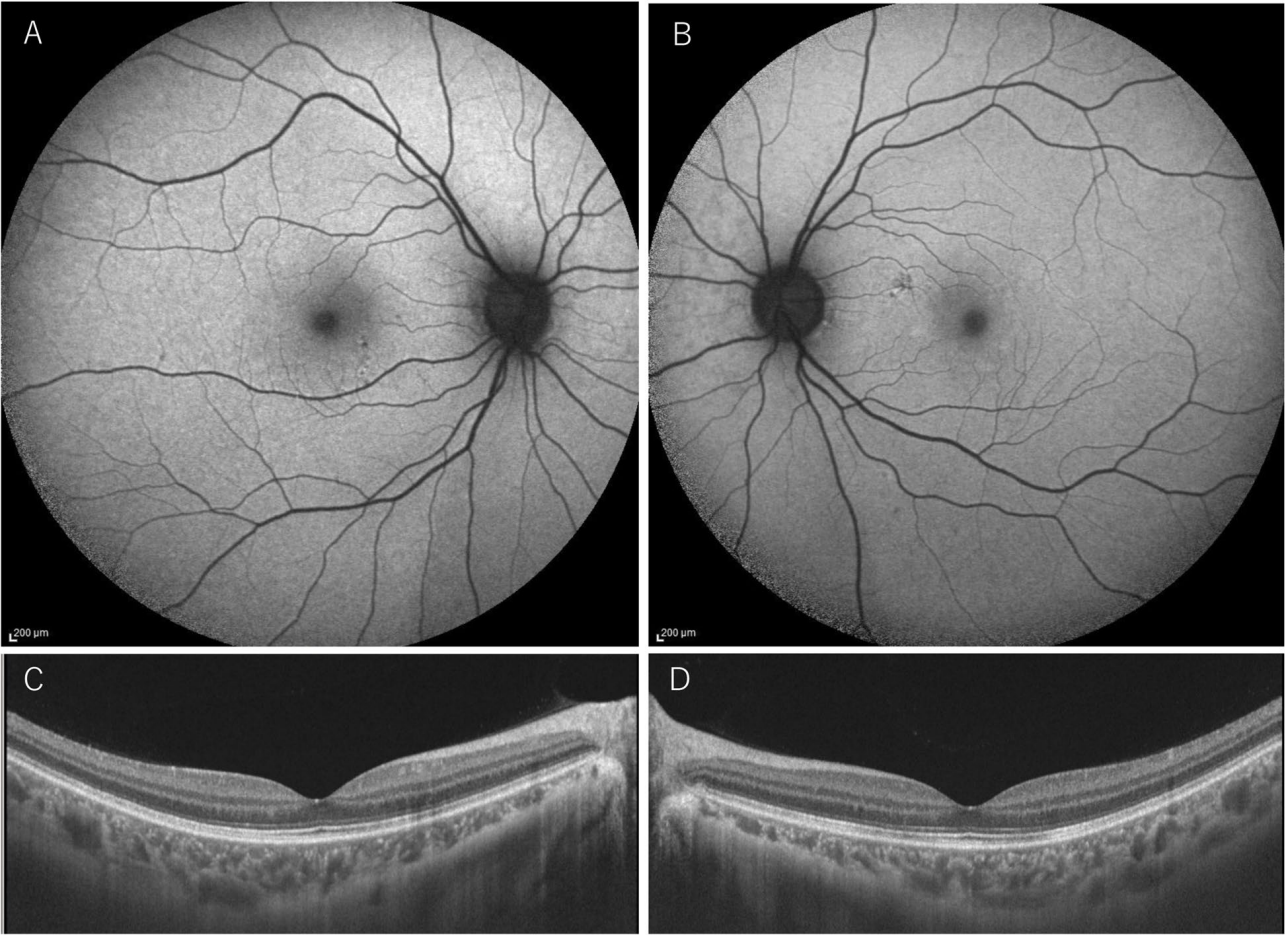
FAF and OCT after steroid pulse therapy. FAF revealed that hyperautofluorescence had disappeared OD **A** and OS **B**. OCT revealed improvement of the outer retinal layer OD **C** and OS **D**

Five days after the steroid pulse treatment, the hyperfluorescence on FAF disappeared, and the outer retinal layer on OCT improved (Fig. 4). The patient’s BCVA recovered to 1.0 OU, and oral PSL was tapered and stopped 5 weeks later. Twelve months after the end of treatment, the patient did not show any recurrences, and no retinal scar lesions have developed. She did not receive a third COVID-19 vaccination.

## Discussion and conclusions

COVID-19 vaccines such as Comirnaty and Spikevax are RNA vaccines that induce strong antibody production against severe acute respiratory syndrome coronavirus 2 (SARS-CoV-2). Higher antibody titers than those after infection with SARS-CoV-2 have been reported after two doses of the vaccine [1]. However, because this vaccine has a strong immunostimulatory effect, adverse events in various organs—including immune thrombocytopenic purpura [2, 3], autoimmune liver diseases [4, 5], Guillain–Barré syndrome [6, 7], IgA nephropathy [8, 9], and inflammatory arthritis [10]—have been reported.

Uveitis can also develop as an adverse event after COVID-19 vaccination, and several case series have been reported [11, 12]. Although most cases have been anterior uveitis, various types of uveitis with posterior segment inflammations—including VKH disease [13], acute zonal occult outer retinopathy [14], multifocal choroiditis [15], and multiple evanescent white dot syndrome [16]—also have been reported. We observed this case of bilateral APMPPE-like panuveitis developed 6 days after the inoculation of the COVID-19 vaccination. Although we cannot rule out the possibility that the disease occurred independent of the vaccine, we strongly suspect that it was linked to the vaccine because of the short time between vaccination and the onset of the disease and the atypical findings for a known uveitis.

APMPPE is a white dot syndrome characterized by white placoid lesions, generally within the posterior pole. It was first reported by Gass in 1968 [17]. Typically, the disease does not produce anterior segment inflammation, or if it does, it is minimal. FA shows hypofluorescence in the early phase and hyperfluorescence in the late phase corresponding to the white placoid lesions on the fundus.

The following point in our case was consistent with APMPPE: first, bilateral white placoid lesions that were hypofluorescent in the early phase and hyperfluorescent in the late phase on FA, and second, sharply marginated hypofluorescent dots of various sizes throughout the mid-venous and late phases on ICGA. However, the patient demonstrated relatively severe anterior segment inflammation that persisted, as well as hyperautofluorescence on FAF and impairment of the outer retinal layer on OCT that were not seen at the initial visit but appeared over 1 week later. These findings were atypical for APMPPE. SRD may also be seen in APMPPE; however, it is unusual to be present in both eyes.

The impairment of the outer retinal layer in the focal area of placoid lesions found by OCT is consistent with findings in APMPPE. Goldenberg et al. proposed a classification of OCT findings seen in APMPPE into 4 distinct stages [18]: At stage 1, demonstrates dome-shaped elevation with disruption of the photoreceptor junction is found. Approximately 2 weeks later, at stage 2, a distinct separation between the photoreceptor junction and the retinal pigment epithelium (RPE) is developed. At stage 3 accentuated RPE hyperreflectivity and union of the RPE and photoreceptor junction, and stage 4 is the resolution phase. The outer retinal layer impairment observed in this patient corresponds to stage 2 or stage 3; however, the outer retinal layer impairment presented in extensive areas that may have been related to the subjective symptom of dark vision was quite different from findings seen in typical APMPPE. Autoimmune retinopathy (AIR) is a representative disease that acutely causes extensive outer retinal layer impairment. The treatment strategy for AIR has not been established; however, some cases have been reported in which steroid pulse therapy has been effective. In this case, extensive outer retinal layer impairment likely was caused by an autoimmune mechanism; therefore, steroid pulse therapy was performed. Of course, it is undeniable that the patient might have recovered without treatment.

The characteristic FAF findings in APMPPE, such as a mixed pattern of a central zone of hypoautofluorescence with a surrounding stippled border of hyperautofluorescence seen in areas of lesions that later become scars, have been reported [19]. In this patient, FAF revealed isoautofluorescence at the onset of the disease. However, during the disease’s progression, FAF showed hyperautofluorescence consistent with the area of impairment of the outer retinal layer seen on OCT. Then, it recovered to isoautofluorescence, leaving no hypoautofluorescent lesions, following corticosteroid treatment. Hyperautofluorescence indicates dysfunction of the outer segment of the retina and/or RPE, and it may be a good indicator of therapeutic intervention.

There have been several reports of APMPPE associated with COVID-19 infection or vaccination. Olguín et al. reported a case of APMPPE developing 2 weeks after a COVID-19 infection [20]. Atas et al. reported a case of APMPPE after a first COVID-19 vaccination. In these cases, all findings from slit-lamp examination, funduscopy examination, FA, and OCT were typical of APMPPE. Additionally, the case reported by Atas demonstrated a good visual prognosis without any treatment, which is also a typical clinical course for APMPPE [21]. In contrast, in our case, APMPPE was the most appropriate diagnosis upon the initial visit based on multimodal imaging; therefore, the patient was provided no medication at first. However, the subsequent significant impairment of her outer retinal layer led to steroid pulse therapy. As a result, the impairment of the outer retinal layer recovered on OCT, the hyperautofluorescent lesions on FAF disappeared, and the patient’s BCVA recovered to 1.0 OU. No visual dysfunction remained; however, we do not know whether retinal scar lesions leading to visual dysfunction would have developed if the patient had been provided with no medication.

APMPPE’s pathogenesis is unknown, but it is often preceded by a viral prodrome [22]. Gonome et al. reported a case of APMPPE with granulomatous lesions after influenza vaccination [23]. Activation of the immune system by vaccination or viral infection may be related to APMPPE’s development.

We observed a case of APMPPE-like panuveitis after COVID-19 vaccination featuring atypical findings for APMPPE. COVID-19 vaccination may not only trigger the onset of the disease but also produce an atypical feature of the disease by strongly activating the immune system. Considering appropriate treatment in each case is necessary.

## Supplementary Information

**Additional file 1: Supplementary Figure.** FA and ICGA of right eye in the early and late phases from the initial visit. FA revealed hypofluorescence in the early phase (A) and hyperfluorescence in the late phase (B) corresponding to cream-white lesions. ICGA revealed sharply marginated hypofluorescent dots of various sizes throughout the mid-venous (C) and late phases (D).

## Data Availability

The datasets used and/or analysed during the current study available from the corresponding author on reasonable request.

## Abbreviations

COVID-19: A coronavirus disease 2019
APMPPE: Acute posterior multifocal placoid pigment epitheliopathy
OCT: Optical coherence tomography
SRD: Serous retinal detachment
FA: Fluorescein angiography
ICGA: Indocyanine green angiography
PSL: Prednisolone
BCVA: Best corrected visual acuity
FAF: Fundus autofluorescence
VKH: Vogt–Koyanagi–Harada
RPE: Retinal pigment epithelium
AIR: Autoimmune retinopathy
