# Supplementary material for: A case of APMPPE-like panuveitis presenting with extensive outer retinal layer impairment following COVID-19 vaccination
Source: BMC Ophthalmol. 2023 May 24;23:233. doi: 10.1186/s12886-023-02978-2 (PMC10206362; doi:10.1186/s12886-023-02978-2)
Supplement: Supplementary file 1 — Additional file 1: Supplementary Figure. FA and ICGA of right eye in the early and late phases from the initial visit. FA revealed hypofluorescence in the early phase (A) and hyperfluorescence in the late phase (B) corresponding to cream-white lesions. ICGA revealed sharply marginated hypofluorescent dots of various sizes throughout the mid-venous (C) and late phases (D). [file 12886_2023_2978_MOESM1_ESM.pdf]

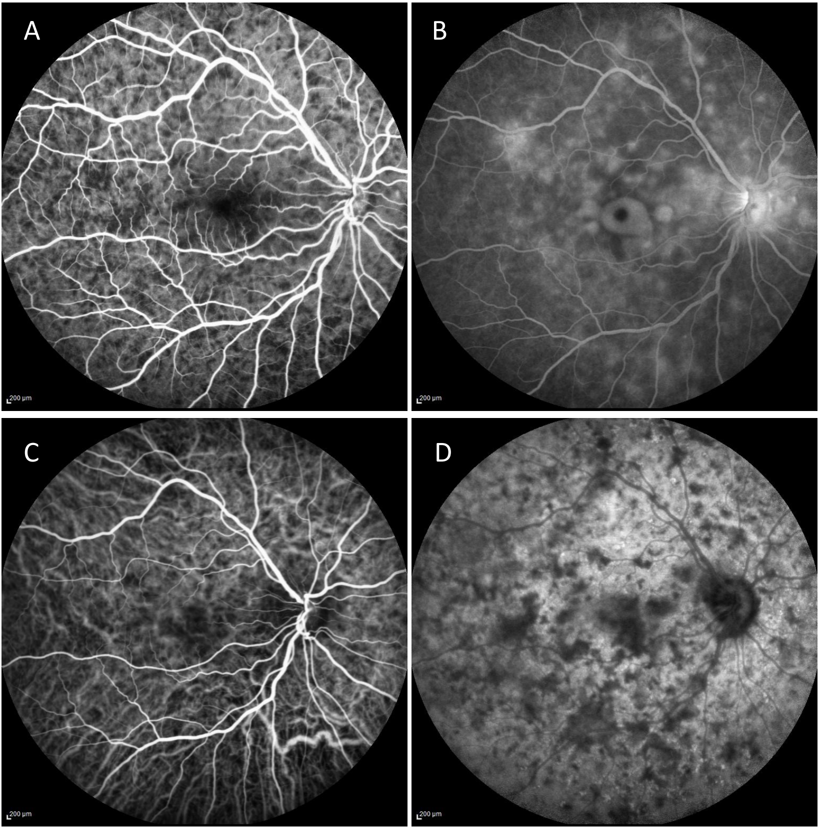

Supplementary Figure. FA and ICGA of right eye in the early and late phases from the initial visit

FA revealed hypofluorescence in the early phase (A) and hyperfluorescence in the late phase (B) corresponding to cream-white lesions. ICGA revealed sharply marginated hypofluorescent dots of various sizes throughout the mid-venous (C) and late phases (D).
